# Effect of Seasonal Variation on Relapse Rate in Patients With Relapsing-Remitting Multiple Sclerosis in Saudi Arabia

**DOI:** 10.3389/fneur.2022.862120

**Published:** 2022-03-14

**Authors:** Seraj Makkawi, Ammar Aljabri, Ghassan Bin Lajdam, Ammar Albakistani, Abdulrahman Aljohani, Suhail Labban, Razaz Felemban

**Affiliations:** ^1^College of Medicine, King Saud Bin Abdulaziz University for Health Sciences, Jeddah, Saudi Arabia; ^2^King Abdullah International Medical Research Center, Jeddah, Saudi Arabia; ^3^Department of Medicine, Ministry of the National Guard-Health Affairs, Jeddah, Saudi Arabia

**Keywords:** multiple sclerosis, seasonal variation, vitamin D, relapse, neuroinflammation

## Abstract

Multiple sclerosis (MS) is becoming a global subject of study in which some demographic variations are thought to be correlated with its activity. Relapsing-remitting multiple sclerosis (RRMS) is the most common demyelinating disorder, characterized by periods of exacerbating attacks, followed by partial or complete remission. Several factors might play a role in disease progression and relapse frequency, such as vitamin D, ultraviolet B radiation, estrogen levels, smoking, obesity, and unhealthy lifestyles. In this study, we identified the relationship between seasonal variation and relapse rate and correlated the latter with sex, age, and vitamin D levels in patients with RRMS in Jeddah, Saudi Arabia. We retrospectively collected data from 182 RRMS patients between 2016 and 2021. A total of 219 relapses were documented in 106 patients (58.2 %). The relapse per patient ratio showed a sinusoidal pattern, peaking in January at a rate of 0.49 and troughed in June at a rate of 0.18. There was no difference in relapse rates between men and women (*p* =0.280). There was a significant negative correlation between vitamin D levels and relapse rate (*r* = −0.312, *p* =0.024). Therefore, the relapse rate was higher during the winter and was correlated with low vitamin D levels. However, relapses are likely multifactorial, and more population-based studies are needed to understand the role of environmental variables in MS exacerbation. A better understanding of this relationship will allow for improved treatment and possibly better prevention of relapse.

## Introduction

Multiple sclerosis (MS) is a demyelinating disease of the central nervous system (CNS) characterized by autoimmune inflammation and neurodegeneration (1). The exact etiology of the disease is yet to be determined, but many potential risk factors have been identified, such as vitamin D deficiency and Epstein-Barr virus infection (2, 3). MS is characterized by recurrent episodes of acute inflammation and focal demyelination in the CNS, better known as relapse. Relapse is defined as episodic exacerbation of neurological impairment, which can be both reversible and irreversible (4). Relapse episodes commonly occur in patients with relapsing-remitting MS, the most common phenotype of MS (4). A relapse is marked by periods of exacerbations that last for 24 h or more, during which the symptoms typically worsen, followed by periods of remission and resolution of inflammation (5). It is estimated that the number patients with MS increased from 2.1 million in 2008 to 2.3 million in 2013 worldwide (6). Based on an annualized report between 1980 and 2008, the relapse rate was estimated to be between 0.27 and 1.66 per year (4). In Saudi Arabia, the prevalence of MS cases has significantly increased from 25/1,00,000 in 1998 to 40/1,00,000 in 2008 (7, 8).

The clinical progression of MS changes with seasons (9, 10). This can be attributed to seasonal variations in different physiological parameters, including vitamin D, which plays an important immunological role. It is essential for the activation of lymphocytes, proliferation of T-helper cells, tissue-specific lymphocyte homing, and production of specific antibodies important for the suppression of autoimmune diseases (11). Ultraviolet B-rays (UVB) is the primary source of vitamin D in humans (12). UVB amount is higher in warmer months of the year (13). This might justify the findings of different studies suggesting reduced clinical activity of MS during the summer (9, 10). However, there is increasing evidence that the effect of UVB on MS risk and pathogenesis is independent of vitamin D. In one experiment, scientists independently tested the effects of ultraviolet (UV) radiation and vitamin D on experimental autoimmune encephalomyelitis mouse models of MS. Vitamin D helped to suppress disease progression due to subsequent hypercalcemia, while UV radiation succeeded in achieving the same result, but not due to a change in calcium levels (14). Other studies have considered seasonal variation as an independent risk factor because of the positive latitude gradient for the prevalence of MS (15). In particular, a study conducted in Australia found that the incidence increased by 9.55% per degree increase in latitude (16). Moreover, migration to sunny climates in childhood reduces the risk of MS (17). The relationship between UV and UV-induced vitamin D levels remains unclear, and more studies are needed to investigate the independent effect of UV radiation on the suppression of autoimmune diseases.

Many risk factors that increase the risk of developing MS and/or exacerbate the disease have been identified. Intensive research has been conducted to determine the role of vitamin D in the development of MS. Findings suggest that low vitamin D serum levels are associated with increased activity and the incidence of MS (18). Vitamin D supplementation was proven to reduce disease activity during low UVB radiation periods of the year such as winter, and early spring (19). A prospective cohort study, which included 145 patients with relapsing-remitting MS in Australia, concluded that vitamin D supplementation decreased the relapse rate by 12% (19). For patients with normal vitamin D levels, the relapse rate decreased by 57% with vitamin D supplementation (20). Moreover, some studies have suggested a significant role for vitamin D in the embryological development of the CNS and in decreasing the prevalence of MS in adulthood (21). Nielsen et al. investigated the correlation between low vitamin D levels at birth and the risk of developing MS in a population-based case-control study in Denmark (21). Residual dried blood spot samples from neonates who later developed MS were analyzed and compared with a control group of the same birthdate and sex (21). The authors observed low vitamin levels in patients who developed MS (21).

Infection is a suggested environmental risk factor for the development of MS (22). Epstein-Barr virus (EBV), a member of the herpes virus family that infects 90% of the general population, is the most commonly associated virus with MS (22, 23). EBV primarily infects children and is typically latent. However, in adolescence or adulthood, EBV can cause an illness known as infectious mononucleosis (IM) (24). It has been suggested that patients with a history of IM have a 2–3 times higher risk of developing MS (25). On the other hand, the risk of developing MS in patients without IM is 15 times lower compared to those who have IM (3).

Furthermore, many studies have found a link between an unhealthy lifestyle and susceptibility to developing MS. Smoking and coffee drinking, for example, are found to increase the risk of developing MS (26). Alcoholism and smoking have also been found to reduce the efficacy of drugs used for MS (27, 28). However, in 2018, a study conducted across three major cities in Saudi Arabia found that a high level of sun exposure, consumption of fruits, and drinking coffee during primary and secondary schools was protective against MS (29). Moreover, it was suggested that significant consumption of fast food predisposes individuals to MS (29).

Physiological factors can also contribute to the development of MS. One example is the postmenopausal decline in estrogen levels, which is thought to be associated with an increased frequency of relapses. Because estrogen has neuroprotective functions, its decline could lead to higher exacerbation rates (30, 31). Furthermore, an inverse correlation between melatonin levels and seasonality of MS relapse has been evident in recent studies. However, clinical evidence for such a correlation is limited (32, 33).

Seasonal variation has been shown to play an important role among the risk factors for increased frequency of relapse. Seasons with higher amounts of sunshine, such as summer, were found to correlate with a low frequency of disease activity (9, 10). UVB radiation, vitamin D intake, and altitude are the main factors responsible for the potential role of seasonal variation in the pathogenesis of MS (9, 10). However, despite many studies reaching similar findings, conflicting results have been reported. For instance, a study from Wales observed a peak frequency of relapse during summer, particularly in June (34). Another study conducted in Japan found no significant difference in relapses between winter and summer (35). A consistent and universal understanding of the impact of seasonal variation on relapse frequency is missing. Clarification of seasonal variation in MS relapse in more regions could contribute to the ongoing discussion on mechanisms for MS relapses and a possibly better understanding of this potential association. This study aimed to evaluate the impact of seasonal variation in different climate variables and vitamin D levels on patients with RRMS in a tertiary hospital in Jeddah, Saudi Arabia.

## Materials and Methods

This retrospective study was conducted in the neurological department of King Abdulaziz Medical City (KAMC), Jeddah. The study included all patients diagnosed with relapsing-remitting MS according to the revised McDonald criteria between January 2016 and January 2021. Only clinical relapses with worsening symptoms lasting more than 24 h were included. Consequent relapses in the same patient were considered as two different relapses if the period between them exceeded one month. Pseudo-relapses and relapses in which patients had positive urinalysis were excluded. One hundred and eighty two patients met our inclusion criteria. Consecutive sampling was used to obtain a representative sample of these patients. The data were collected by the investigators once the study was approved. All cases of relapse were recorded by direct observation of the selected medical records of patients from 2016–2021 on the BESTCare 2.0A system at King Abdulaziz Medical Center, Jeddah. A data collection sheet was used to gather information on the number of relapses and the date of onset. Data were also collected on age, sex, magnetic resonance imaging results, and disease-modifying treatment (DMTs) of the participants. The patients were divided into two age groups (>40 and <40) since relapses are age depended. Each relapse was categorized into one of the 12 months and four seasons of the year according to the time of occurrence. The outcome variable was the relapse rate *per season*, which was estimated by dividing the number of relapses by the study duration in days and multiplying the result by 365 days. The readings of the vitamin D analysis, in addition to the date of taking the vitamin D analysis, were collected from the BestCare 2.0 system using a data collection sheet based on the available data. Moreover, because vitamin D analysis were randomly taking between follow ups, each relapse date was linked to the nearest vitamin D analysis date to assess the correlation between vitamin D levels and the relapse rate.

### Analytical Analysis

Data analysis was performed using the Statistical Package for the Social Sciences (SPSS) version 26 (36). Age, sex, age at diagnosis, illness duration, and body mass index (BMI) were included as covariates. These tables were used to illustrate some descriptive measures. Frequency and percentages were used to depict the qualitative data. The mean and standard deviation were used to describe the parametric quantitative data. The median and range values were used to represent non-parametric quantitative data. Descriptive data were displayed using graphs. A line graph was used to show the variation in the number of relapses per patient in each month of the year. In addition, a clustered column chart was used to depict the relationship between monthly relapses, projected temperatures (maximum and minimum), and rainfall. For inferential statistics, Pearson's correlation was used to evaluate the correlation between the relapse rate and vitamin D levels. In addition, a chi-square test was conducted to investigate the association between relapses and categorical variables such as age and sex.

## Results

### Patients

This study included 182 patients, of whom 123 (67.6%) were women. Patients were divided into two age classes: 111 (61%) patients, who were <40 years of age and 71 (39%) patients, who were more than 40 years of age. In terms of the MS variant, all included patients had the relapsing-remitting MS variant. The mean disease duration was seven years (range-23 years).

### Relapses

**Figure 1:**
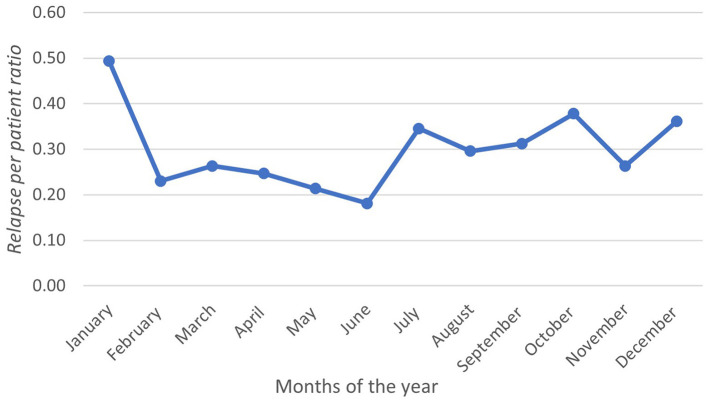
Line graph showing relapse per patient ratio in each month, as calculated.

**Figure 2:**
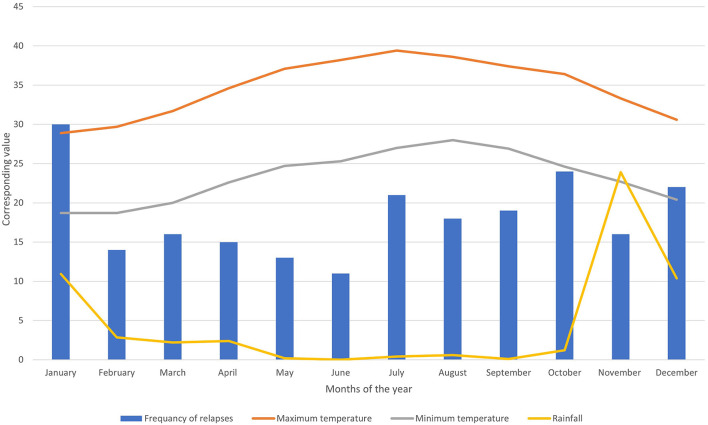
Bar chart showing the frequency of relapses per month combined with three-line graphs showing max. and min. temperature and rainfall.

During the observation period, 219 relapses were documented in 106 (58.2%) patients. Of the 106 patients, 75 (70.7%) were women (Table 1). The relapse/patient ratio showed a sinusoidal pattern, reflecting the seasonal variation. The ratio peaked in January at a rate of 0.49 and troughed in June at a rate of 0.18 (Figure 1). A graph depicting the frequency of relapses per month with the climate variables is shown in Figure 2.

**Table 1:** Patient characteristics.

**Variable**	**Total, N (%) 182 (100.0)**	
Gender, N (%)		
	Female	123 (67.6)
	Male	59 (32.4)
Age, N (%)		
	≤ 40	111 (61.0)
	>40	71 (39.0)
Patients with events, N (%)		106 (100.0)
	Female	75 (70.8)
	Male	31 (29.2)
Current Age, median (min-max)		
	38 (59)	
Age of diagnosis, median (min-max)		
	29 (49)	
Disease duration, median (min-max)		
	7 (23)	
BMI, mean (SD)		
	22.17 (4.77)	

### Clinical Variables and Relapses

A chi-square test of independence showed that relapses were not found to be more likely in one age group (<40 years and >40 years), X2 (1, *N* = 182) = 0.040a, *p* = 0.842. Additionally, there was no significant association between sex and relapse, *X*2 (1, *N* = 182) = 1.166a, *p* = 0.280 (Table 2).

**Table 2:** Model for clinical factors associated with relapse in patients with MS.

**Variable**	**Chi square value**	***P* = value**
**Age group** <40 years >40 years	0.040^a^	0.842
**Gender** Male Female	1.166^a^	0.280

a *means less than 20% of cells have expected count less than 5*.

### Vitamin D Levels and Relapses

Out of 182 patients, only 135 (74.12%) had their vitamin D levels recorded. The mean vitamin D level was 57.70 ± 28.55 (Table 3). Patients were divided into three categories based on their vitamin D levels: Normal (≥50 nmol/L), insufficient (30 to 50 nmol/L), and deficient (<30 mol/L). 69 patients (51.1%) had normal vitamin D levels (Table 4). Higher vitamin D levels were observed in females and patients aged 40 or younger. However, the difference was not statistically significant among all comparisons. 68 patients (50.37) had more than one relapse and the vitamin D levels were slightly lower for each consecutive relapse. Relapse was more likely to occur in patients with lower vitamin D levels. Pearson's correlation showed a significant negative correlation between vitamin D levels and relapse rater = −0.312, *p* = 0.024.

**Table 3:** Model for vitamin D levels based on relapse.

**Relapse number**	**Frequency**	**Mean**	**Std. deviation**
Vitamin D at Relapse 1	52	50.696	36.5161
Vitamin D at Relapse 2	30	47.367	30.6443
Vitamin D at Relapse 3–9	39	47.082	36.088
Total mean of vitamin D levels	135	57.709	2.457

**Table 4:** Model for vitamin D levels based on gender and age.

		**Normal**	**Insufficiency**	**Deficiency**	**Total**
Gender	Female	47	41	9	97
	Male	22	13	3	38
	Total	69	54	12	135
	*p* value	0.613			
Age	>40	22	21	6	49
	≤ 40	47	33	6	86
	Total	69	54	12	135
	*p* value	0.425			

## Discussion

The burden of MS has increased significantly over the last two decades, particularly in Saudi Arabia. This study aimed to determine the relationship between seasonal variation and relapse rates in patients with relapsing-remitting MS in King Abdulaziz Medical City, Jeddah, Saudi Arabia. We observed a seasonal pattern in the relapse rate. Most relapses occurred in the winter months. Our findings also suggest that the number of relapses is inversely related to the average rainfall and overall temperature. Moreover, there was a significant negative correlation between the relapse rate and vitamin D levels, possibly highlighting its protective role against relapse. The study included two age groups (>40 and <40) due to relapses being age dependent and majority of disease onset before 40 were of the relapsing remitting type (37). Our study is the first to demonstrate the effect of seasonal variation on relapse rates of MS in the Middle East.

A seasonal pattern of MS relapse has been noted in multiple studies conducted across different geographical locations. Some studies reported a peak in the number of relapses in spring and/or summer (35, 38, 39). This pattern of seasonal variation is supported by a meta-analysis (40). However, most of these studies were conducted in North America and Europe. Others have observed a nadir in relapse rate, mostly occurring in late summer or autumn (41, 42). Our observed pattern of seasonality is similar to that of a recent Danish population-based cohort study that reported a lower rate of relapse in the summer months (43). Furthermore, in a population-based Australian study conducted in southern Tasmania, a peak in the relapse rate was observed during winter (42). However, two studies did not observe a seasonal pattern of MS relapse (44, 45). Both of these were small Southern European studies, where there was less variation in climate.

An association between UVB radiation and the relapse rate in MS has been previously reported. Although the mean vitamin D level was normal, patients with deficiency had more relapses. The mean vitamin D level also decreased with each consecutive relapse. Similar to our findings, several studies have demonstrated the role of vitamin D in decreasing relapse rates in patients with relapsing-remitting MS (18, 19). This has been attributed to the role of UVB radiation in vitamin D production. More hours of sunshine exposure during the summer allows for higher production of 25 hydroxyvitamin D possibly explaining the nadir in the relapse rate during the warm months of the year. Vitamin D supplementations were found to reduce frequency of relapses, with greater effect on those with lower baseline levels of vitamin D (46). Moreover, it has been proposed that infections, particularly upper respiratory tract infections (URTI), are involved in the exacerbation of MS (47). Interestingly, URTIs rates were higher in individuals with lower vitamin D levels and exhibited a similar seasonal pattern to MS relapse, with more infections occurring in the winter months (42). Vitamin D has been shown to affect the function of the innate immune system, which in turn could modify the rate of infection and relapse in people with MS.

This is the first retrospective study to be conducted in our region. Being confined to a single geographic location minimizes other environmental confounding factors implicated in the disease course. The study included climate variables and allowed for multiple relapses in the same individual to be considered, highlighting individual variations in the relapse rate. It also included vitamin D levels, linking them to the exacerbation rate and other meteorological data.

The study included a limited number of participants compared to previous studies that studied the role of meteorological variables in the relapse rate. However, owing to the lower prevalence of MS in our region compared to the West, the study results are valid for answering the research questions. Furthermore, being a single-center study minimizes the role of other environmental confounders, and it does not allow for an independent examination of climate variables with relapse rates. Such variations in geographical locations would allow for stronger associations between meteorological data and relapse rates. Finally, some relapses were excluded from the analysis because an accurate date could not be determined. Such patients were mostly those with a single visit, incomplete relapse characteristics, and lack of follow-up. Only if relapses with uncertain dates showed a different seasonal pattern than those with a confirmed date would it bias the conclusions of the study.

In conclusion, this study included different meteorological data that were used to identify any seasonal pattern in the rate of MS relapse. The relapse rate peaked during winter. A better understanding of this relationship will allow for improved treatment and possibly, better prevention of relapse. Relapses are likely multifactorial, and more population-based studies are needed to understand the role of environmental variables in MS exacerbation.

## Data Availability Statement

The original contributions presented in the study are included in the article/supplementary material, further inquiries can be directed to the corresponding author.

## Ethics Statement

The studies involving human participants were reviewed and approved by Institutional Review Board - King Abdullah International Medical Research Center. Written informed consent for participation was not required for this study in accordance with the national legislation and the institutional requirements.

## Author Contributions

SM contributed to conceptualization, supervision of the project, writing, and editing the manuscript. AAlja, GL, AAlb, AAljo, and SL contributed to writing the manuscript and data collection. RF contributed to supervising and editing the manuscript. All authors contributed to the article and approved the submitted version.

## Conflict of Interest

The authors declare that the research was conducted in the absence of any commercial or financial relationships that could be construed as a potential conflict of interest.

## Publisher's Note

All claims expressed in this article are solely those of the authors and do not necessarily represent those of their affiliated organizations, or those of the publisher, the editors and the reviewers. Any product that may be evaluated in this article, or claim that may be made by its manufacturer, is not guaranteed or endorsed by the publisher.
